# Combined DNA Analysis from Stool and Blood Samples Improves Tumor Tracking and Assessment of Clonal Heterogeneity in Localized Rectal Cancer Patients

**DOI:** 10.1177/15330338241252706

**Published:** 2024-05-20

**Authors:** Thomas Parigger, Franz Josef Gassner, Stephan Drothler, Christian Scherhäufl, Alexandra Hödlmoser, Lena Schultheis, Aryunni Abu Bakar, Florian Huemer, Richard Greil, Roland Geisberger, Lukas Weiss, Nadja Zaborsky

**Affiliations:** 1 Department Laboratory of Immunological and Molecular Cancer Research-Salzburg Cancer Research Institute, Cancer Cluster Salzburg, Salzburg, Austria; 2 Department of Internal Medicine III with Haematology, Medical Oncology, Haemostaseology, Infectiology and Rheumatology, Oncologic Center, Paracelsus Medical University, Salzburg, Austria; 3 Department of Biosciences, Paris-Lodron-University Salzburg, Salzburg, Austria

**Keywords:** colorectal cancer, next generation sequencing, liquid biopsy, mutation analysis, tumor heterogeneity

## Abstract

**Objectives:** In this study, stool samples were evaluated for tumor mutation analysis *via* a targeted next generation sequencing (NGS) approach in a small patient cohort suffering from localized rectal cancer. **Introduction:** Colorectal cancer (CRC) causes the second highest cancer-related death rate worldwide. Thus, improvements in disease assessment and monitoring that may facilitate treatment allocation and allow organ-sparing “watch-and-wait” treatment strategies are highly relevant for a significant number of CRC patients. **Methods:** Stool-based results were compared with mutation profiles derived from liquid biopsies and the gold standard procedure of tumor biopsy from the same patients. A workflow was established that enables the detection of *de-novo* tumor mutations in stool samples of CRC patients *via* ultra-sensitive cell-free tumor DNA target enrichment. **Results:** Notably, only a 19% overall concordance was found in mutational profiles across the compared sample specimens of stool, tumor, and liquid biopsies. **Conclusion:** Based on these results, the analysis of stool and liquid biopsy samples can provide important additional information on tumor heterogeneity and potentially on the assessment of minimal residual disease and clonal tumor evolution.

## Introduction

With over 1.9 million new cases per year, colorectal cancer (CRC) accounts for the second highest cancer-related death rate worldwide and thus poses a major health problem.^1^ Survival of patients with CRC is inversely related to the disease stage at diagnosis and screening programs have been shown to reduce incidence and mortality rates of CRC.^2^

Currently, colonoscopy and tumor biopsy represent the gold standards for CRC diagnosis and CRC mutation analysis, respectively. However, the technical feasibility, tumor heterogeneity, and potential clonal evolution represent challenges for tumor characterization using DNA from such locally constrained tumor biopsies.^3^

A relatively new and anatomically more independent option for non-invasive cancer screening, detection, prognosis, and surveillance is based on the analysis of cell-free DNA (cfDNA) and particularly circulating tumor DNA (ctDNA) in the blood (liquid biopsy). cfDNA displays an extensive complexity that derives from a multiplicity of DNA origins such as host cells, pathogens, and microbes. Nevertheless, due to the tremendous progress in cfDNA extraction efficiency, sequencing technology, and bioinformatics procedures, liquid biopsies can be deciphered with ever-increasing accuracy.^4^ Further, ctDNA may provide a more holistic mutation profile of the tumor mass compared to biopsies and has the potential to detect metastasized tumor cells at distant locations.^5^ Studies showed that a high quantity of cfDNA as well as the mere detection of ctDNA in CRC patients correlates with a shorter overall survival (OS) and can therefore be used as a prognostic marker.^6,7^ Additionally, sequential ctDNA-based mutation detection can be applied for therapy monitoring, clonal evolution, and minimal residual disease (MRD) detection.^8^ However, the potential low abundance of ctDNA in the blood and the general age-dependent accumulation of premalignant mutations represent challenges of liquid biopsy testing, especially in early stage CRC.

Alternatively, stool DNA has also been tested for tumor mutation detection and despite its composition of various different DNA sources such as normal gastrointestinal cells, microbiota, and nutriments, it may serve as an easily accessible DNA source for the detection of gastrointestinal tumor mutations. A recent study comparing the detection rate of *KRAS* (KRAS proto-oncogene, GTPase) mutations not only in blood and tumor tissue but also in stool, found a higher overall agreement between stool versus tissue (84.9%) than between blood versus tissue (77.4%) in CRC patients.^9^ Another study comparing the detection of known mutations in blood and stool of CRC patients concluded that stool may even provide a better source for mutation testing than plasma.^10^

Thus, it can be speculated that mutation analysis in blood and especially stool of CRC patients using an ultra-sensitive next generation sequencing (NGS) approach could allow the detection of CRC mutations with unprecedented sensitivity and specificity. Furthermore, by covering a larger gene panel than previous studies, blood and especially stool mutation analysis might serve as a highly sensitive surveillance and therapy monitoring approach for CRC patients.

In this study, plasma and stool samples of 12 patients with localized rectal cancer (T3-4 N0-2 M0) were analyzed for tumor mutations utilizing a ctDNA kit covering 197 cancer-associated genes. These medically highly relevant genetic regions encode variant loci that are listed in various mutation databases (COSMIC, TCGA, ExAc, dbSNP, 1000 Genomes, SnpEff). Additionally, mutation analysis of tumor biopsies from the same patients was performed using a gene panel that covers 4800 disease-associated genomic regions derived from various mutation databases (HGMD, OMIM, ClinVar). The main aim of these analyses was to provide more information about the utility and additional benefit of tumor mutation detection in stool samples and the concordance with plasma and tumor biopsy samples.

## Materials and Methods

### Patient Criteria

Patients had to have histologically confirmed adenocarcinoma of the rectum with locoregional, non-metastatic disease. All patients underwent standard neoadjuvant chemoradiotherapy at the Department of Internal Medicine III of the Paracelsus Medical University (Salzburg, Austria) followed by surgery.

### Study Design

Biopsy tissue, blood, and stool samples were collected from treatment-naïve locally advanced rectal cancer patients (n = 12) and additionally from two CRC patients of the same cohort post-therapy (time interval up to eight weeks after chemoradiotherapy completion). All patients were treated with capecitabine 825 mg/m^2^ twice a day orally during radiotherapy interval. Clinical staging was carried out by magnetic resonance imaging (MRI) of the pelvis, computed tomography (CT) scans of the thorax and abdomen as well as endorectal ultrasound. Mutation analysis of stool samples and cfDNA derived from blood was conducted and compared to variants detected in tumor tissue biopsies and in one patient (Pat ID 1462) to tumor resection tissue.

### Plasma ctDNA Sequencing:

Plasma (9 ml) was isolated from peripheral blood collected in Streck vials (cat. no. 346064-6, Streck, La Vista, USA) by centrifugation (1600 g, 10 min and 6000 g, 10 min) and was subsequently stored at −80°C. 2.8-3.7 ml of plasma was used to isolate cell-free DNA (cfDNA) *via* the AVENIO^TM^ cfDNA Isolation Kit (Roche, Basel, Switzerland). Sequencing libraries were established by using the ctDNA Library Prep Kit and ctDNA Enrichment Kit in combination with the Surveillance panel (cat. no. 08061084001, Roche, Basel, Switzerland, cat.: 08061084001). The panel covers 197 genes and regions including all exonic (±20 bp flanking regions, without 3′/5′ UTR) regions of 11 genes: *APC* (APC regulator of WNT signaling pathway), *BRAF* (B-Raf proto-oncogene, serine/threonine kinase), *BRCA1* (BRCA1 DNA repair associated), *BRCA2* (BRCA2 DNA repair associated), *DPYD* (dihydropyrimidine dehydrogenase)¸ *KIT* (KIT proto-oncogene, receptor tyrosine kinase), *KRAS*, *NRAS* (NRAS proto-oncogene, GTPase), *PDGFRA* (platelet derived growth factor receptor alpha), *TP53* (tumor protein p53), *UGT1A1* (UDP glucuronosyltransferase family 1 member A1) covering single nucleotide variants (SNV), and InDels; as well as exonic (±20 bp flanking regions, without 3′/5′ UTR) regions of the genes *EGFR* (epidermal growth factor receptor), *ERBB2* (erb-b2 receptor tyrosine kinase 2), and *MET* (MET proto-oncogene, receptor tyrosine kinase) covering SNV, InDel and amplifications; as well as exonic (±20 bp flanking regions) and intronic regions of three genes: *ALK* (ALK receptor tyrosine kinase), *RET* (ret proto-oncogene), *ROS1* (ROS proto-oncogene 1, receptor tyrosine kinase) covering SNV, InDel and Fusion mutations. Additionally, the ctDNA Surveillance panel covers 180 cancer-associated genetic hotspots within exonic (without 3′/5′ UTR) regions covering SNV. Mutations in splice sites, 3′ UTR and 5′UTR regions can be covered within the targets that include ±20 bp flanking regions. Final libraries were quality checked using the Bioanalyzer TapeStation (cat. no. G2991BA, Agilent, Santa Clara, USA, cat.: G2991BA) and loaded onto a NextSeq 550 instrument (Illumina, San Diego, USA, cat. no. SY-415-1002) (1.5 pM, paired-end, 150 bp, 15% PhiX). The average number of read pairs and mapped reads constituted 38.9 × 10^6^ (27 × 10^6^-58 × 10^6^, SD 9.5 × 10^6^) and 73 × 10^6^ (54 × 10^6^-106 × 10^6^, SD 17.5 × 10^6^), respectively. The unique median sequencing depth across all plasma samples was 2930 (2731-5054, SD 1549). Sequencing data were analyzed by the company Roche (Basel, Switzerland) using their proprietary analysis software. Non-synonymous and synonymous SNV, InDels, amplifications, and Fusions within the respective func.refGene region of the individual targets (described above) were reported in order to accurately compare the different sample specimens. All automatically detected mutations in any sample were manually checked in all corresponding sample specimens of the same patient.

### Stool DNA Quality Assessment

PCR reactions were conducted, using 10 ng of stool DNA (except for sample 1364_2 from which 3.4 ng was used) as a template and primers which are either specific for a region of the human gene *SAMHD1* (SAM and HD domain-containing deoxynucleoside triphosphate triphosphohydrolase 1) (primer: Fwd CTACCTCGGATGTTCTTCAGCAG 10 µM, Rev AATAGGCTGCCAATACTCCTTGG 10 µM, Eurofins Genomics, Luxembourg, Luxembourg;) or the regions *V3* and *V4* of the bacterial gene *16S* (16S rRNA gene) (primers: Fwd 5′ TCGTCGGCAGCGTCAGATGTGTATAAGAGACAGCCTACGGGNGGCWGCAG, Rev 5′ GTCTCGTGGGCTCGGAGATGTGTATAAGAGACAGGACTACHVGGGTATCTAATCC, *16S* Metagenomic Sequencing Library Preparation, Illumina, San Diego, USA, cat. no. 15044223), for which well-established amplification and detection protocols exist within the research facility, were conducted. *SAMHD1* PCR reaction: 10 µl Phusion High-Fidelity PCR Master Mix (Thermo Scientific, Waltham, USA, cat. no F531L), 1 µl of each primer, 2 µl DNA template (98°C 30 s, 98°C 10 s, 65°C 20 s, 72°C 20 s, 72°C 5 min, 30 cycles). *16S* PCR reaction: 10 µl Kappa HiFi HotStart Ready Mix (Roche, Basel, Switzerland, cat. no. 07958960001), 4 µl of each primer, 2 µl DNA template (95°C 3 min, 95°C 30 s, 55°C 30 s, 72°C 30 s, 72°C 5 min, 25 cycles). DNA concentration of the specific PCR products was measured on the Bioanalyzer TapeStation and used to estimate the human and bacterial DNA fractions within the stool samples.

### Stool Sequencing

Stool was collected in stool sample devices (Norgen, Thorold, Canada) and DNA was isolated using the QIAamp Fast DNA Stool Mini Kit (QIAGEN, Hilden, Germany, cat. no. 51604). 1.5 µg of isolated DNA was mechanically sheared into 150-200 bp fragments using the Covaris M220 system (duty factor: 20%, peak incident power: 50, cycles per burst: 200, time: 320 s, temperature: 20°C) (Covaris, Woburn, USA, cat.no. 500295F) in order to make the stool DNA compatible for the AVENIO^TM^ ctDNA workflow. Stool DNA fragments were quality checked using the Bioanalyzer TapeStation and subsequently used as input (50 ng) for ctDNA Library Prep Kit and the ctDNA Enrichment Kit in combination with the Surveillance panel. Final libraries were quality checked *via* the Bioanalyzer TapeStation and sequenced on a NextSeq 550 instrument (1.5 pM, paired-end, 150 bp, 15% PhiX). The average number of read pairs and mapped reads constituted 36.7 × 10^6^ (14.1 × 10^6^-58 × 10^6^, SD 10.7 × 10^6^) and 38.2 × 10^6^ (10.5 × 10^6^-108 × 10^6^, SD 31 × 10^6^), respectively. The unique median sequencing depth across all stool samples was 229 (91-2378, SD 675). Sequencing data were analyzed by the company Roche using their proprietary analysis software with the following variations from default checkpoints: percentage of reads aligned to human genome: 15% (default 90%), unique molecular depth: 90 (default 500). From three samples that initially failed in the analysis, two (Pat ID 1360_2, 1364) were re-sequenced and successfully analyzed using newly extracted DNA, which passed the human PCR threshold (2.5 ng/µl). One sample (Pat ID 1349), which failed the analysis and did not reach the PCR threshold in the second DNA extraction, could be analyzed *via* an in-house established analysis pipeline. In brief, reads were trimmed using Trimmomatic (v 0.33, default settings),^11^ Trimmed sequencing reads were aligned to the hg38 reference genome using bwa mem (v0.7.12-r1039, arXiv: 1303.3997, default settings and -M option) and the resulting sam files were processed with picard-tools MarkDuplicates (v2.21.9, default settings, https://broadinstitute.github.io/picard/, accessed 03.03.2023) to remove duplicates. Sam-bam conversion, sorting, and indexing were performed by samtools (v1.10).^12^ Bam file processing was performed including the following operations: RealignerTargetCreator, IndelRealigner, BaseRecalibrator (hg38.dbsnp151.common_all_20180418 as knownSites database), PrintReads (all from GATK v3.7.0, default settings).^13^ NM tag repair was performed using samtools calmd. For assessment of somatic variants, a pileup file was generated (samtools mpileup –B –q 1). VarScan (v2.4.4)^14^ and the pipeline proposed by Koboldt et al.^15^ were used for variant calling (–min-var-freq 0.001). SNP (single-nucleotide polymorphism) variants were annotated using ANNOVAR's (v2017-07-17)^16^ table_annovar.pl script and the following databases: refGene, avsnp150, clinvar_20200316, cosmic92, exac03, icgc28. Non-synonymous and synonymous SNV, InDels, amplifications, and Fusions within the respective func.refGene region were reported in order to accurately compare the different sample specimens. All automatically detected mutations in any sample were manually checked in all corresponding sample specimens of the same patient. Two stool samples, although being successfully sequenced and analyzed, had to be excluded due to a cross-contamination within these samples detected during quality control.

### Colorectal Tumor Sequencing

DNA was extracted from formalin fixed paraffin embedded (FFPE) embedded colorectal tumor biopsies or, for one patient, from resected tumor tissue using the AllPrep DNA/RNA FFPE Kit (Qiagen, Hilden, Germany, cat. no. 80234). Matched germline DNA was isolated from either peripheral blood (DNeasy Blood and tissue Kit, Qiagen, Hilden, Germany, cat. no. 69504) or buccal swaps (Maxwell 16 FFPE Tissue LEV DNA Purification Kit, Promega, Madison, USA, cat. no. AS1130). Because the AVENIO^TM^ Tumor Tissue targeted kit was not available at the time, which covers the same genetic regions as the AVENIO^TM^ ctDNA Library Prep Kit used for plasma and stool samples, the SureSelect^TM^ XT HS Target Enrichment Kit in combination with the SureSelect^TM^ XT HS Focused Exome capture library (Agilent, Santa Clara, USA, cat. no. G9702A; 5190-7787) was used for tissue and resection mutation analysis. The Focused Exome panel targets exonic regions (±10 bp flanking regions) of 4800 disease-associated genes. Mutations in splice sites, 3′UTR, 5′UTR, and intronic regions can be covered within the ±10 bp flanking regions and the 50 bp region adjacent to designed capture probes. 45-200 ng of DNA was used as input material for library preparation. Libraries were quality checked *via* Bioanalyzer TapeStation and sequenced on a NextSeq 550 instrument (1.2 pM, paired-end, 150 bp, 1% PhiX).

Mean read number was 51.9 × 10^6^ (20.9-114 × 10^6^, SD 23.4 × 10^6^) for tumor samples and 13.8 × 10^6^ (6.5-22.8 × 10^6^, SD 3.8 × 10^6^) for germline samples (Supplemental Table 1). Mean coverage of mutated loci was 171.2 reads (10-567) in FFPE tissue samples which is adequate for detecting tumor mutations in tissues that consist a high tumor cell fraction. Mean number of variant reads was 24.9 reads (3-101). Manually called mutations in FFPE tissue samples were based on automatically called identical mutations in blood and stool samples. Lowest allelic frequency was 9% and 0.5% for automatically and manually called mutations, respectively.

Fastq files were adaptor trimmed using the Agilent Genomics NextGen Toolkit (AGeNT) 2.0.5 (trimmer-2.0.3.jar -xt) and quality checked using the FASTQC software (v0.11.9).^17^ Trimmed sequencing reads were aligned to the hg38 reference genome using bwa mem (v0.7.12-r1039, arXiv: 1303.3997, default settings and M -C options) and the resulting sam files were processed with AGeNT 2.0.5 (locatit-2.0.5.jar -PM:xm,Q:xq,q:nQ,r:nR -S -i -r) to remove duplicates. Sam-bam conversion, sorting, and indexing were performed by samtools (v1.10).^12^ Bam file processing was performed including the following operations: RealignerTargetCreator, IndelRealigner, BaseRecalibrator (hg38.dbsnp151.common_all_20180418 as known Sites database), PrintReads (GATK v3.7.0, default settings).^13^ NM tag repair was performed using samtools calmd. For assessment of somatic variants, first a pileup file was generated (samtools mpileup –B –q 1) for each sample pair (germline, tumor). VarScan (v2.4.4)^14^ and the pipeline proposed by Koboldt et al.^15^ was used for variant calling (–min-tumor-freq 0.02, –max-normal-freq 0.05). Variant filtering was performed using the fpfilter script (https://github.com/dkoboldt/varscan/blob/master/VarScan.v2.4.4.description.txt, accessed 03.03.2023) with the following arguments: –dream3-settings 1, –min-var-freq 0.02, –min-var-avgrl 70, –min-ref-avgrl 70, –min-var-basequal 10, –max-rl-diff 1. The filtered SNP variants were annotated using ANNOVAR's (v2017-07-17)^16^ table_annovar.pl script and the following databases: refGene, avsnp150, clinvar_20200316, cosmic92, exac03. Non-synonymous and synonymous variants and InDels covered by the targeted SureSelect^TM^ XT HS Focused Exome capture library (Agilent, Santa Clara, USA) were reported in order to accurately compare the different sample specimens.

### Statistical Testing

For the comparison of allelic frequencies as well as number of mutations within different sample specimens, a statistical t-test was performed. All available paired patient samples at the third Medical Department of the University Hospital Salzburg at the time point of analysis were used for this study without conducting an a priori power calculation for estimating the sample size.

## Results

Twelve unselected, consecutive patients who were all treated with neoadjuvant chemoradiotherapy with capecitabine for locally advanced rectal cancer and who were willing to participate in this pilot study were included. The prodrug capecitabine is activated by thymidine phosphorylases into the cytotoxic moiety fluorouracil, which inhibits DNA-, RNA-, and protein synthesis. The patient cohort consists of mainly male patients (75%) with a mean age at diagnosis of 61 years. All but one patient had clinical tumor stages T3 with varying amounts of positive regional lymph nodes (N1-2) but no distant metastases (M0) (Table 1).

**Table 1.** Patient Characteristics.

Patient ID	Age (years)	Sex	Rectum localization	Distance from anal verge (cm)	Exulceration	cTNM first diagnosis	Tumor grading
1360	64	M	Middle	7	Yes	T3N1M0	2
1364	61	F	lower	2	No	T3N1M0	2
1345	65	M	middle	7	Yes	T4N2M0	1
1349	49	M	middle	5	No	T3N1M0	2
1385	51	F	lower	3	Yes	T3N1M0	2
1402	65	M	middle	5	No	T3N1M0	2
1462	70	M	lower	3	Yes	T3N1M0	2
1471	60	M	lower	1	Yes	T3N0M0	2
1477	50	M	lower	2	Yes	T3N1M0	2
1478	59	M	middle	6	Yes	T3N2M0	2
1484	79	F	lower	0	No	T3N0M0	2
1486	60	M	lower	2	No	T3N0M0	2

### DNA Extracted from Stool is Applicable for Targeted Ultra-Sensitive NGS Mutation Analysis

**Figure 1.**
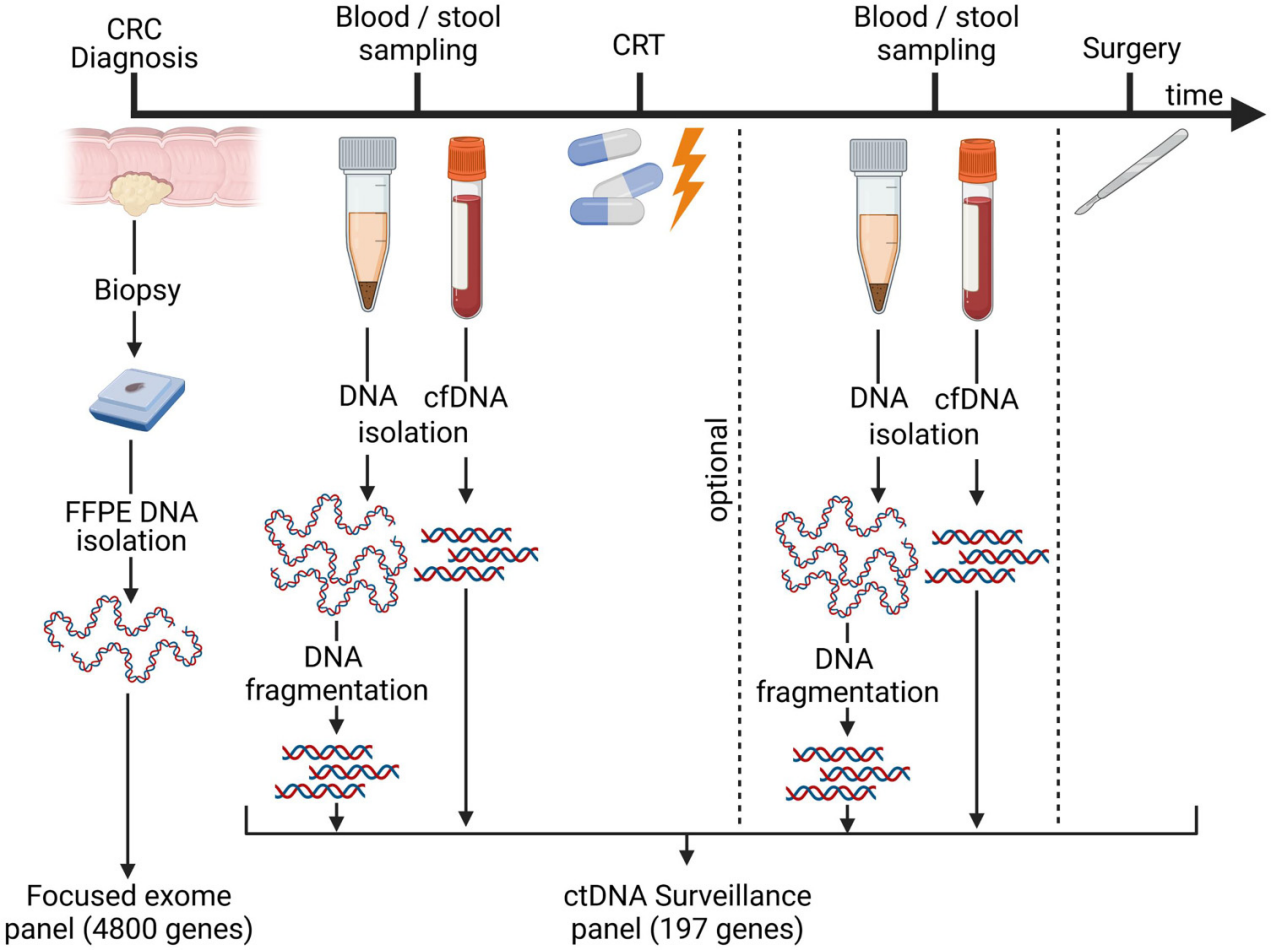
Sampling process. Timeline of biopsy, blood, and stool specimen collection. Tumor biopsies were taken before any treatment was initiated. Blood and stool samples were collected before neoadjuvant chemoradiotherapy (CRT) and optionally in the interval between CRT completion and curative surgery (up to eight weeks after CRT completion). Biopsy samples were analyzed using the SureSelect^TM^ XT HS-focused exome panel comprising 4800 genes and regions, whereas blood and stool samples were investigated using the AVENIO^TM^ ctDNA surveillance panel covering 197 genes and regions. FFPE: formalin-fixed, paraffin-embedded; cfDNA: cell-free DNA, ctDNA: cell-free tumor DNA, CRT: chemoradiotherapy. Figure created with Biorender.com.

In order to investigate the utility of tumor mutation detection in stool, stool samples were collected prior to therapy start from 12 rectal cancer patients and analyzed *via* an ultra-sensitive ctDNA NGS kit. Simultaneously, plasma was collected from the same treatment-naïve patients and was analyzed together with the stool samples in a one-batch process. From selected patients, plasma (2.8-3.7 mL) and stool samples were additionally collected after chemoradiotherapy in order to study the stool-based detection of MRD (Supplemental Table 1). The tumor tissue was derived from FPPE biopsies sampled at the time of CRC diagnosis or, in one case, from surgically resected tumor tissue (Figure 1).

The utilized high-sensitivity AVENIO^TM^ ctDNA enrichment kit is a research-use-only product and is intended for the investigation of plasma-derived ctDNA of late-stage cancer patients (in particular lung cancer and CRC patients).

In total, 12 stool samples could be successfully analyzed for tumor mutations. Initially, three stool samples did not fulfill the default quality criteria of the analysis software pipeline due to a very low fraction of reads mapping to the human genome or a generally low unique read depth. In order to investigate the human DNA fraction in stool samples, the randomly selected human gene *SAMHD1* was PCR amplified. This method uncovered that a PCR product yield <2.5 ng/µl identifies stool samples that do not contain a sufficient amount of human DNA to be analyzed successfully. Therefore, DNA was re-extracted from the initially failed as well as from new stool samples and libraries were established and sequenced only when the threshold of 2.5 ng/µl of human PCR product was reached. All stool samples above the PCR threshold could be successfully analyzed.

All 14 libraries established from plasma cfDNA were successfully sequenced in-house and analyzed via the proprietary Roche analysis pipeline.

### Stool DNA Yields Higher Mutational Allelic Frequencies Compared to Plasma Despite Lower Human Library Fractions

**Figure 2.**
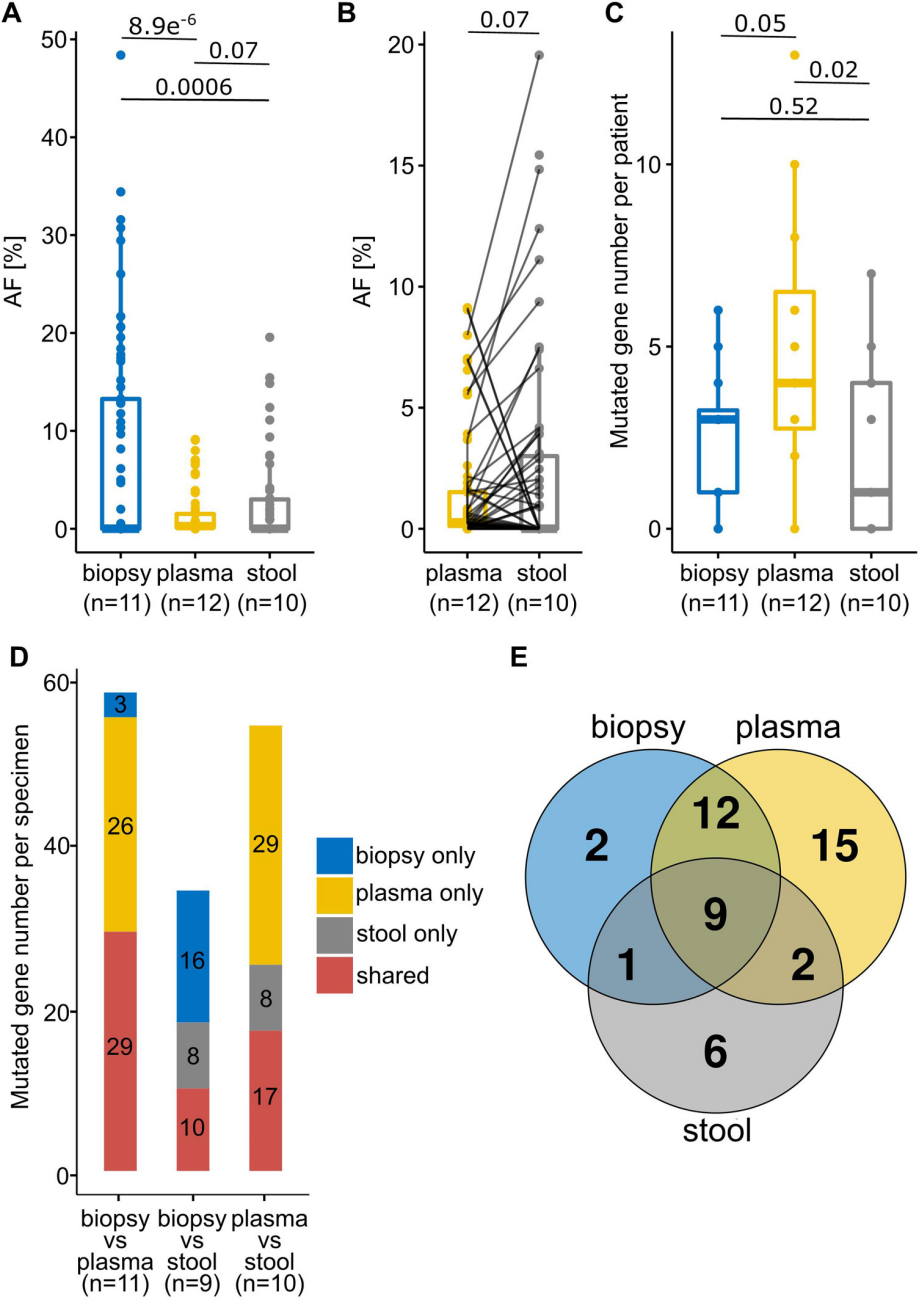
Mutational allelic frequencies, numbers, and direct comparisons of identified mutations in different specimens. (A) Allelic frequencies (AF) of mutations identified in different sample specimens. (B) Comparison of AF of detected mutations in paired plasma and stool samples derived from the same patients. (C) Number of identified mutations per patient within different sample specimens. (D) Number of shared and non-shared mutations in the same patient across different specimen comparisons. (E) Number of intersecting mutations in different specimens across all patients (n = 9) from whom all three specimens were analyzed. Only mutations that are covered by both the SureSelect^TM^ (biopsy) and AVENIO^TM^ (plasma and stool) panel are depicted. Numbers above lines in graphs (A–C) represent p-values.

Overall, the analyses detected a mean mutation number per sample of 20.5 (12-43, n = 11), 7.6 (0-13, n = 14), and 3.9 (0-16, n = 12) in tumor biopsy, plasma, and stool samples, respectively (Table 2, Supplemental Table 1). However, to be able to compare the sample specimens analyzed by different kits, only shared genetic regions (105 genes) of the two sequencing panels for tumor biopsies (SureSelect^TM^) and plasma/stool (AVENIO^TM^) were considered (Supplemental Table 2). All automatically detected mutations in any specimen were manually checked in all corresponding specimens of the same patient. As expected, the mean allelic frequency (AF) of detected mutations was significantly higher in tumor biopsies (8.4%, 0.34-48%) compared to the other specimens (plasma p = 8.9 × 10^−6^, stool p = 0.0006) (Figure 2A, Supplemental Table 1). When comparing sequencing metrics, plasma, and stool samples show a median unique read depth of 2930 and 229.5 and mean on-target rate of 72.5% and 54.7%, respectively (Table 2; Supplemental Table 1). Interestingly, despite a lower human library fraction, we found a higher mean AF of mutations in stool (2.5%, 0.9-22.8%) compared to plasma cfDNA samples (1.3%, 0.09-9.1%) (p = 0.07) (Figure 2B; Supplemental Table 1). Examination of the median numbers of detected mutations per patient, revealed that the highest values are found in plasma samples (4), followed by tissue biopsies (3) and finally stool samples (2) (Figure 2C).

**Table 2.** Sequencing Metrics of Individual Sequencing Approaches.

Specimen	Sample number	Mean mutations/ sample	Gene-panel (size)	Mean sequencing reads (×10^6^)	Median unique read depth	Mean on-target rate (%)	Mean mutation coverage
Tumor biopsy	11	20.5	SureSelect^TM^ XT HS (4800 genes and regions)	51.9	171.2	92.8	24.9
Resection	1	30	SureSelect^TM^ XT HS (4800 genes and regions)	95.1	267	92.6	34
Plasma	14	7.6	AVENIO^TM^ ctDNA Surveillance (17 genes / regions, 180 hotspots)	77.6	2930	72.5	45
Stool	12	3.9	AVENIO^TM^ ctDNA Surveillance (17 genes / regions, 180 hotspots)	73.4	229.5	54.7	23.4

Sequencing metrics of all samples derived from specific specimens analyzed with SureSelect^TM^ XT HS Focused Exome or AVENIO^TM^ ctDNA Surveillance approach. The SureSelect^TM^ XT HS Focused Exome approach contains non-synonymous exonic (+-10 bp flanking regions) variants. The specimens sequenced *via* the AVENIO^TM^ ctDNA Surveillance approach include synonymous and non-synonymous mutations within the exonic (+-20 bp flanking regions) region.

Comparing the detection of identical mutations across paired sample specimens revealed that 29 mutations were shared between biopsy and plasma, whereas 10 or 17 mutations were simultaneously found in biopsy and stool or plasma and stool, respectively. However, a significant number of mutations was not shared between individual specimens (biopsy and plasma: 29, biopsy and stool: 24, plasma and stool: 37) (Figure 2D). Strikingly, when comparing the identified mutations of patients of whom all three specimens were successfully analyzed (n = 9), only 9 mutations can be detected that are shared across all specimens (biopsy, plasma, stool) in contrast to 23 mutations which are exclusively found in one specific specimen (Figure 2E).

### Combined Analysis of Different Sample Specimens can Improve Assessment of Tumor Heterogeneity, Clonal Evolution, and MRD

**Figure 3.**
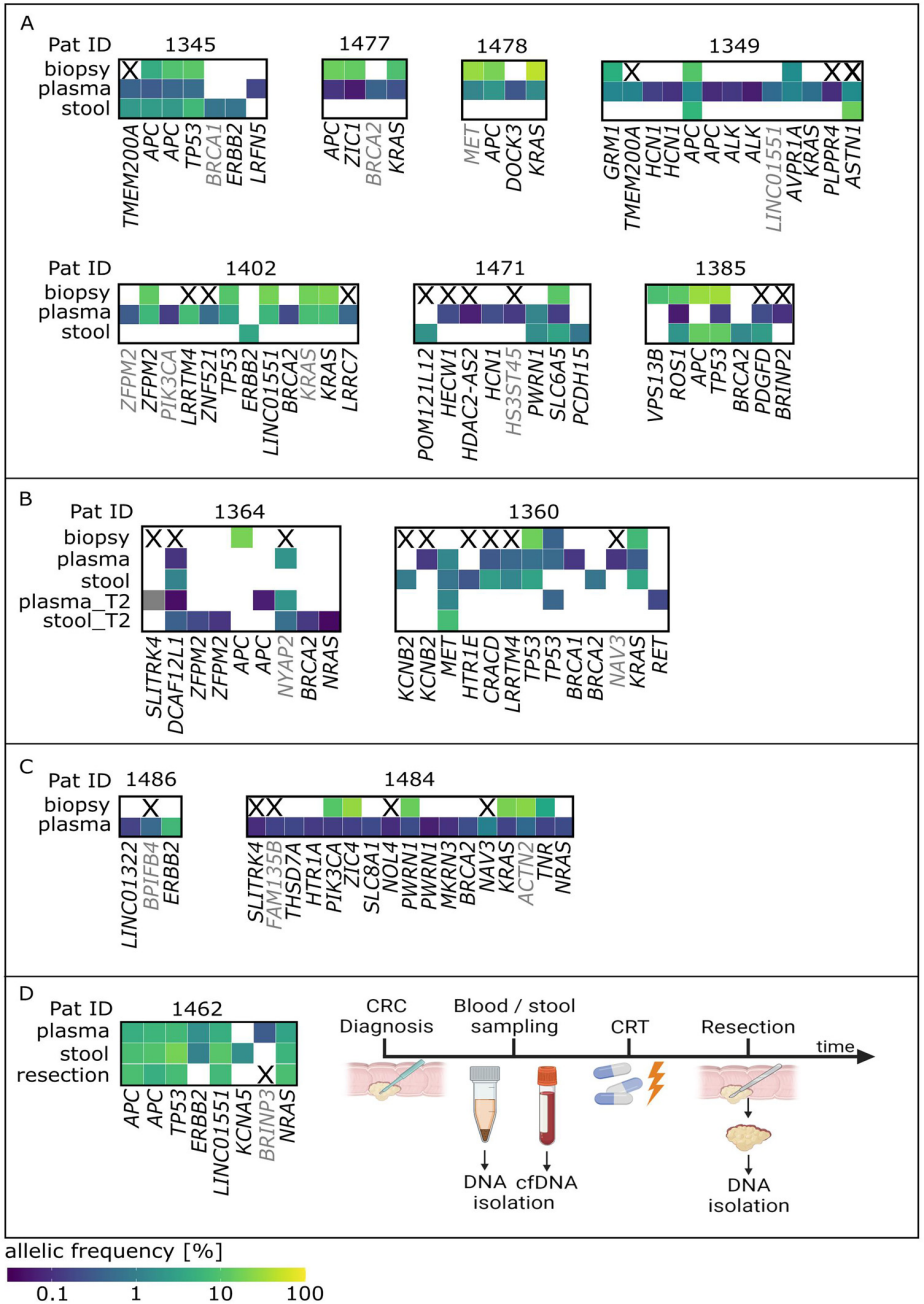
Comparison of identified mutations across different sample specimens in individual patients. (A) Mutational lesions detected in either tumor biopsy, plasma, or stool samples in individual patients. (B) Mutational analysis in patients from whom plasma and stool samples were available before (plasma/stool) and after (plasma_T2/stool_T2) therapeutic intervention. (C) Comparison of variants detected in tumor biopsies and liquid biopsies (plasma) in patients from whom stool mutational analysis was not available. (D) Comparison of mutations in different sample specimens of patient 1462 whose plasma and stool mutation profile is compared to a resection of the whole tumor (resection). Timeline of the course of disease, sample collection, and therapeutic interventions of Pat 1462 are depicted. Genes marked with X in tissue samples are not covered by the SureSelect^TM^ sequencing panel used for biopsy specimen analysis. Gene names represent individual mutational positions within a certain gene. Grey gene names represent synonymous mutations. Color code represents allelic frequency. CRT: chemoradiotherapy; cfDNA: cell-free DNA.

Comparing individual mutations detected in different sample specimens (tumor biopsy, plasma, and stool) of the same patient often revealed distinct mutational profiles. Although there were patients in which several identical mutations (>2) were identified in all three different specimens (Pat ID 1345), this seems to be the exception. Overall, highly varying degrees of mutational concordance were found between different sample specimens. A fraction of patients (Pat ID 1477, 1478, 1402, and 1360) displayed a similarity (≥50%) between biopsy and plasma, other patients share similar mutation profiles in plasma and stool (Pat ID 1385 and 1462) or across biopsy and stool (Pat ID 1385). However, the majority of patients reveal a highly variable mutational concordance across different sample specimens (Figure 3A–D).

From two patients, additional plasma and stool samples were provided after therapeutic intervention (Pat ID 1364 and 1360). The post-chemoradiotherapy (but pre-operative) samples of patient 1364 revealed two previously undetected mutations in plasma (*SLITRK4* (SLIT and NTRK like family member 4), *APC*) and even four newly detected variants (2× *ZFPM2* (zinc finger protein, FOG family member 2), *BRCA2*, *NRAS*) in the stool sample. Similarly, in patient 1360 novel (*RET*) and already recognized mutations (*MET*, *TP53*) were detected after chemoradiotherapy (Figure 3B).

Other patient samples (Pat ID 1486 and 1484) also show a discrepancy of mutations detected in biopsy and plasma. None of the mutations detected in plasma of patient 1486 could be identified in the respective biopsy sample of the same patient. Similarly, the majority of mutations detected in the plasma sample of patient 1484 were not identified in the tumor biopsy (Figure 3C; Supplemental Table 1). *Vice versa*, despite a relatively high AF, only two mutations were exclusively detected in tumor biopsies (*VPS13B* (vacuolar protein sorting 13 homolog B), AF 0.09, Pat ID 1385 and *APC*, AF 0.2, Pat ID 1364), compared to 15 and 6 mutations that were detected only in plasma or stool, respectively.

Moreover, in patient 1462 plasma and stool samples revealed several shared CRC-related mutational lesions. Unfortunately, not enough tumor biopsy material was available for analysis. Therefore, a mutation analysis of available resected tumor material, which was sampled post-chemoradiotherapy, was conducted and revealed several mutations that were already detected in plasma and stool before treatment. However, one mutation (*ERBB2*) found in both plasma and stool and another mutation (*KCNA5* (potassium voltage-gated channel subfamily A member 5)) observed only in stool could not be detected in the resected tumor tissue (Figure 3D; Supplemental Table 1).

Notably, several mutations (5× *BRCA2*, 4× *ERBB2*, 2× *NRAS*, 2× *HCN1* [hyperpolarization-activated cyclic nucleotide-gated potassium channel 1]) were detected more than once in plasma or stool samples across all patients but never in tumor biopsies.

## Discussion

This study provides the first evidence of tumor mutation detection from stool specimens using an ultra-sensitive ctDNA target enrichment kit originally designed for liquid biopsies. In order to minimize potential bias due to inter-patient variability, the presented study was exclusively performed in patients with rectal cancer, therefore with tumors in near proximity to the anus. The average transit time of stool in the colon can vary widely between individuals and is influenced by factors such as diet, age, and health status.^18^ Stool samples can only be taken after defecation, thereby leading to differential times of exposure of tumor-derived nucleic acids in the stool. Depending on the primary tumor site, this leads to substantially longer exposures in the case of tumors in the ascending colon when compared to rectal tumors.

Here we show that this stool-based approach holds a great potential for the detection of tumor mutations in CRC patients. However, individual stool DNA samples comprise variable fractions of human DNA and typically a rather high amount of bacterial DNA of gut microbiota, which minimizes the human library fraction. This high bacterial DNA contamination in specific stool DNA samples might be the major cause for the failure of the mutation analysis. Therefore, a quality control for stool specimens based on the PCR-based estimation of the human DNA fraction of stool samples is proposed. After the introduction of this quality checkpoint, all samples found eligible for library preparation could be successfully analyzed. A general adaptation of the library preparation kit and -analysis pipeline specifically for stool samples could potentially improve its sensitivity further. Increasing the read depth of stool library samples or the specific enrichment of human stool DNA could further improve the mutation detection limit in stool samples. Notably, many tumor mutations were detectable in stool samples with a higher AF compared to plasma ctDNA and a crucial amount of mutations were exclusively detected in the stool samples of the patients. This high AF in stool samples likely derives from the very close proximity of the colorectal tumor to the stool, whereby tumor cells are easily shed into the colon lumen in a high quantity. By increasing the read depth of stool samples, stool-based mutation detection could reach a very high sensitivity, which is the prerequisite for an effective screening approach. By improving this workflow, a highly attractive and non-invasive screening strategy could potentially be established that might help identify mutations found in precancerous lesions and thereby lower the chance of malignant transformation through timely intervention. Of course, such a screening approach would have to be tested in a separate study focusing on a precancerous and early stage CRC patient cohort.

Noteworthy, a stool-based in vitro diagnostic test (Cologuard®) has already been approved by the Food and Drug Administration (FDA) for CRC and advanced adenoma screening in individuals older than 50 years. Cologuard® includes three different biomarker analyses (KRAS mutations, NDRG4 and BMP3 methylation pattern, and occult hemoglobin) and proved superior to fecal immunochemical tests (FIT) in terms of CRC and advanced precancerous lesion detection.^19^ However, due to the restriction to three genes and stool specimens only, Cologuard® cannot accurately depict tumor heterogeneity.

The additional value of each individual specimen can be appreciated when comparing the overlap of detected mutations in two different paired specimens. The highest percentage (50%) of overlapping mutations between two specimens is found in biopsy and plasma samples, whereas only 29% and 31% of mutations are shared between biopsy and stool or plasma and stool, respectively. 50-70% of all mutations were only identified in a single specimen and therefore would not be detected if only one specimen was used for analysis. Notably, only 19% of mutations are shared when comparing all three specimens, whereas 49% of mutations are exclusively found in one particular specimen but not in any other specimen. These results may reflect a tumor heterogeneity that is not completely exposed *via* the gold standard of tissue biopsy alone, which is confined to a very small tumor area and thus potentially misses spatially heterogeneous tumor mutations. Hence, the analysis of additional plasma and stool samples provides a higher informational content that is not covered by analyzing a single biopsy sample. Further, these results indicate that it is the combinatorial analysis of all available sample materials that provide the highest informational content.

**Figure 4.**
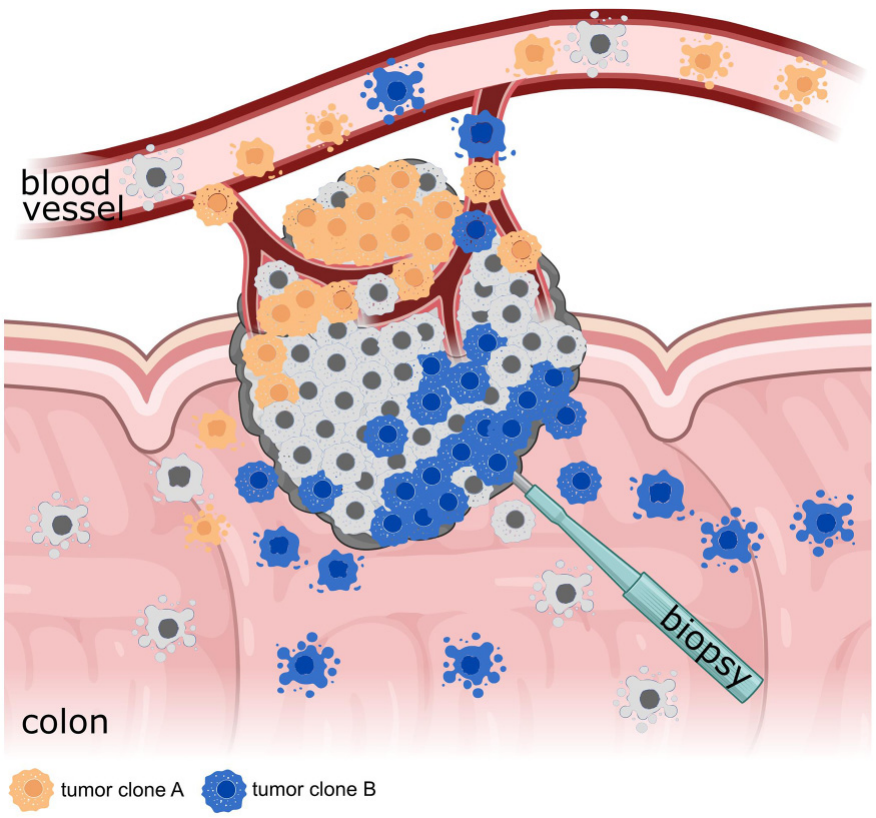
Heterogeneous colorectal tumor. A colorectal tumor consisting of several tumor clones (illustrated by different colors) that grow either in- or outwards of the colorectal lumen. Thereby, different tumor clones are more likely shed either into the blood or the colorectal lumen depending on the growth direction of the tumor clone. Figure created with Biorender.com.

The data display a broad variety of heterogeneity in mutational profiles across biopsy, plasma, and stool samples. It may be hypothesized that the heterogeneity between plasma and stool is also influenced by a distinct dissemination of certain tumor clones into the colon lumen and the circulation. Such a different tumor clone spread might depend on the disease stage, growth direction, and vascularization of the tumor, as well as on the specific mutations of tumor clones that may drive metastasis. Further, the clonal composition of the tumor sections facing either toward the colon lumen or toward highly vascularized tissue may be decisive for the mutational profile found in distinct tissue specimens (Figure 4).^20–22^ Therefore, additional analysis of tumor mutations in plasma and stool may potentially facilitate the assessment of tumor heterogeneity.

A better overview of the tumor heterogeneity and mutational spectrum across different sample specimens by our ultra-sensitive NGS approach covering 197 cancer-associated genes may also help to identify a patient-tailored therapy with the highest probability of a favorable treatment response. *ERBB2* mutations, which have been associated with insensitivities toward EGFR-targeted therapies,^23^ detected only in plasma or stool samples (Pat 1345, 1402, 1486) might help to guide such therapy decisions. Further, *KRAS* and *NRAS* mutations that were only detected in plasma of Pat 1349 and Pat 1484, respectively, might indicate potential primary resistances toward platin- and EGFR therapies.^24–26^

A potential additional value of plasma and stool specimens for disease monitoring was shown in two patients (Pat 1364 and Pat 1360) by revealing variants that have been exclusively recognized post-therapy. Since the half-life of ctDNA in CRC patients is reported to be short (114 min),^27^ the detection of tumor mutations in post-therapy samples is indicative for the presence of residual tumor cells after therapy (plasma_T2, stool_T2). The identification of such variants post-therapy could therefore represent a helpful parameter for the assessment of MRD. All rectal cancer patients in the cohort underwent neoadjuvant chemoradiotherapy followed by curative surgery 6-8 weeks thereafter. However, total neoadjuvant therapy (TNT)—the neoadjuvant employment of radiotherapy or chemoradiation as well as chemotherapy before surgery—has led to increased pathological complete response rates as well as a reduction in the risk of distant metastases in locally advanced rectal cancer.^28–31^ Furthermore, these increased response rates may allow organ-sparing strategies in the case of clinical complete remission following TNT in a growing number of patients with lower rectal cancer. A close follow-up scheme for patients undergoing such a “watch-and-wait” policy is mandatory. Furthermore, it might also assist in early detection of local relapse of colorectal cancer in a non-invasive manner, however, this needs to be investigated in separate future studies.

Novel mutations found in post-therapy plasma and stool of the same patients could potentially point toward a clonal evolution of the tumor cells that might have been driven by the therapeutic intervention. Longitudinal alterations within the tumor mutation profile could be indicative of secondary resistance development against certain treatment regimens and thus can be relevant for further treatment decisions. Newly emerged mutations were detected in *APC* and *NRAS* in plasma and stool samples, respectively, after therapeutic intervention in patient 1364 which could potentially guide further treatment decisions. There is evidence that *APC* mutants have been associated with resistance against fluoropyrimidines such as capecitabine in CRC cell lines.^32^ The aforementioned APC mutation could therefore point toward a secondary capecitabine resistance development that may necessitate a therapy change.

Currently, the potentially confounding effect of clonal hematopoiesis (CH) on liquid biopsy analysis is under investigation. A study investigating colorectal adenocarcinoma patients found 11 of 38 patients (29%) to harbor at least one CH-related mutation.^33^ Since stool specimens are largely free of relevant amounts of blood it is highly unlikely that CH-related mutations can be detected.

## Conclusions

This study provides first evidence that tumor-specific mutations are detectable in different specimens derived from distinct anatomical sites and that the fraction of shared variants is low between the analyzed paired specimens. Thus, complementary plasma and stool mutational analysis can provide valuable information on the tumor heterogeneity, MRD status, and on potential clonal evolution that might be linked to resistance development. This additional information could help physicians to make rational decisions concerning further treatment options.

## Supplemental Material

sj-xlsx-1-tct-10.1177_15330338241252706 - Supplemental material for Combined DNA Analysis from Stool and Blood Samples Improves Tumor Tracking and Assessment of Clonal Heterogeneity in Localized Rectal Cancer Patients Supplemental material, sj-xlsx-1-tct-10.1177_15330338241252706 for Combined DNA Analysis from Stool and Blood Samples Improves Tumor Tracking and Assessment of Clonal Heterogeneity in Localized Rectal Cancer Patients by Thomas Parigger, PhD, Franz Josef Gassner, PhD, Stephan Drothler, MSc, Christian Scherhäufl, MSc, Alexandra Hödlmoser, MSc, Lena Schultheis, MSc, Aryunni Abu Bakar, MSc, Florian Huemer, MD, Richard Greil, MD, Roland Geisberger, PhD, Lukas Weiss, MD, PhD, and Nadja Zaborsky, PhD in Technology in Cancer Research & Treatment

sj-xlsx-2-tct-10.1177_15330338241252706 - Supplemental material for Combined DNA Analysis from Stool and Blood Samples Improves Tumor Tracking and Assessment of Clonal Heterogeneity in Localized Rectal Cancer Patients Supplemental material, sj-xlsx-2-tct-10.1177_15330338241252706 for Combined DNA Analysis from Stool and Blood Samples Improves Tumor Tracking and Assessment of Clonal Heterogeneity in Localized Rectal Cancer Patients by Thomas Parigger, PhD, Franz Josef Gassner, PhD, Stephan Drothler, MSc, Christian Scherhäufl, MSc, Alexandra Hödlmoser, MSc, Lena Schultheis, MSc, Aryunni Abu Bakar, MSc, Florian Huemer, MD, Richard Greil, MD, Roland Geisberger, PhD, Lukas Weiss, MD, PhD, and Nadja Zaborsky, PhD in Technology in Cancer Research & Treatment
